# Editorial: Applications of fungi

**DOI:** 10.3389/fcimb.2026.1832203

**Published:** 2026-04-10

**Authors:** Jesus Perez-Moreno, Saowaluck Tibpromma, Allen Grace T. Niego, Samantha Chandranath Karunarathna

**Affiliations:** 1 Colegio de Posgraduados, Edafología, Texcoco, Mexico; 2 Center for Yunnan Plateau Biological Resources Protection and Utilization & Yunnan International Joint Laboratory of Fungal Sustainable Utilization in South and Southeast Asia, College of Biology and Food Engineering, Qujing Normal University, Qujing, China; 3 Natural Science Department, College of Arts and Sciences, Iloilo Science and Technology University, La Paz, Iloilo, Philippines

**Keywords:** bioactive metabolites, fungal biodiversity, fungal cell factories, metabolic engineering, mycorremediation

The fungal kingdom is among the most diverse and evolutionarily successful lineages on Earth, yet it remains significantly underexplored. Of an estimated 2.5 million species, only about 155,000 have been formally described, revealing a vast reservoir of biological innovation with considerable, largely untapped biotechnological potential. Fungi have historically shaped modern medicine by providing life-saving compounds such as penicillin, cyclosporin, and lovastatin. Beyond these landmark discoveries, their unique physiology, metabolic versatility, and relative ease of cultivation position them as ideal platforms for addressing contemporary challenges in health, agriculture, and environmental sustainability. The Research Topic “*Applications of Fungi*” compiles investigations that demonstrate the multifaceted utility of fungi, spanning molecular mechanisms of adaptation to the development of innovative applications in therapeutics, industry, and bioremediation. Collectively, these contributions underscore a paradigm shift: fungi are increasingly recognized not only as sources of serendipitous discovery but also as engineerable platforms and systematic reservoirs for solutions to global challenges.

A foundational theme woven throughout several contributions is the imperative to understand fundamental fungal biology to unlock its potential for application. The ability of fungi to thrive in diverse, often hostile environments is underpinned by sophisticated regulatory networks. Wangsanut et al. provide a comprehensive review of the AcuK and AcuM transcription factors, a conserved subfamily of fungal-specific regulators. Their analysis across major human pathogens and model saprophytes reveals that these factors act as master switches, orchestrating not only core metabolic pathways such as gluconeogenesis and alternative respiration but also critical stress responses to iron limitation, hypoxia, and host immune interactions. By elucidating the structural basis of AcuK/AcuM heterodimer formation and their role in virulence, this work transcends basic biology, highlighting these transcription factors as high-value targets for the development of novel antifungal therapies. This deep dive into regulatory genomics exemplifies how understanding fungal adaptation is a prerequisite for both combating pathogenic fungi and harnessing the metabolic prowess of beneficial ones.

This principle of deep biological understanding is directly applied in studies that manipulate fungal physiology to enhance product formation. Chutimanukul et al. demonstrate a sophisticated approach to optimizing the cultivation of the medicinal mushroom *Hericium erinaceus*. By moving beyond traditional substrate optimization, they investigate the influence of specific LED light spectra on fungal growth and secondary metabolite production. Their findings, that blue light maximally stimulates both mycelial biomass and the production of metabolites with potent cytotoxicity against colorectal and liver cancer cells, provide a compelling framework for photobiological engineering. This targeted modulation of fungal physiology through environmental cues represents a powerful, non-genetic strategy to enhance the yield of high-value therapeutic compounds. Concurrently, the potential for genetic engineering is powerfully illustrated by Manganyi and Tchatchouang in their mini-review on cannabinoid biosynthesis in engineered fungi. They detail the remarkable biotechnological advancements, including pathway reconstruction, enzyme optimization, and CRISPR-Cas9-mediated genome editing, that are enabling the sustainable production of complex plant-derived compounds such as THC and CBD in fungal platforms. This work positions fungi as versatile microbial cell factories capable of circumventing the agricultural and regulatory limitations of traditional plant cultivation, opening new avenues for the pharmaceutical and nutraceutical industries.

The therapeutic promise of fungi is explored in greater depth through a series of articles focusing on their rich repertoire of bioactive molecules. Wang et al. present a visionary perspective on edible mushrooms as emerging “biofactories” for oral biopharmaceutical delivery. Leveraging their Generally Recognized as Safe (GRAS) status, eukaryotic protein-processing machinery, and natural bioencapsulation capacity, engineered mushrooms offer a transformative platform for the production and delivery of oral vaccines and recombinant proteins. This concept bridges the gap between traditional medicinal use and modern molecular farming. The chemical diversity underpinning these therapeutic effects is further explored in several reviews. Karunarathna et al. provide a focused examination of *Ganoderma*’s antimicrobial properties, detailing the mechanisms by which its polysaccharides, triterpenoids, and phenolic compounds disrupt bacterial integrity and inhibit biofilm formation. Similarly, Karunarathna et al. offer a comprehensive overview of the genus *Fomitopsis*, cataloging its arsenal of bioactive secondary metabolites with antioxidant, anti-inflammatory, and immunomodulatory activities. The immense potential of fungal polysaccharides is a recurring theme, with Kumla et al. providing a broad review of production, extraction, characterization, and multifaceted applications, while Chotmanee et al. delve into the specific immunomodulatory potential of exopolysaccharides (EPs) from Basidiomycetes. Their study demonstrates that EPs from *Schizophyllum commune* and other species can enhance macrophage killing of *Salmonella*, positioning them as potential alternatives to antibiotics in an era of rising antimicrobial resistance. Yang et al. synthesize these concepts by reviewing the innovative applications of medicinal mushrooms in functional foods and nutraceuticals, with a particular focus on the rapidly expanding market for health-boosting beverages. This body of work collectively reinforces the notion that fungi are a prolific source of structurally diverse and therapeutically potent molecules, with applications ranging from direct antimicrobial agents to immune-modulating functional foods.

Beyond human health, this Research Topic highlights the pivotal role of fungi in promoting agricultural and environmental sustainability. The work of Banduwardena et al. addresses the pressing public health challenge posed by insecticide-resistant mosquito vectors. They evaluate the entomopathogenic potential of *Aspergillus niger* and the mycoparasitic *Trichoderma atroviride* against *Aedes aegypti*, the principal vector of dengue. The striking 100% adulticidal mortality achieved by both fungal strains, regardless of the mosquito’s pyrethroid resistance status, underscores the promise of fungal bioinsecticides as a powerful tool for integrated vector management. In the realm of environmental biotechnology, Wimalasena et al. address the pervasive problem of synthetic dye pollution. Isolating filamentous ascomycetes from freshwater environments in Sri Lanka, they demonstrate the remarkable decolourisation capacity of *Lasiodiplodia* species against toxic dyes like Congo Red and Malachite Green. Crucially, this study extends beyond the laboratory by developing a prototype microencapsulation system for real-world mycoremediation, offering a sustainable and cost-effective alternative to conventional physicochemical wastewater treatment. Further contributing to sustainable practices, Thivijan et al. explore the oenological potential of indigenous non-*Saccharomyces* yeasts. Their isolation and characterization of *Hanseniaspora uvarum* from grape skins reveal its capacity to enhance wine quality by improving alcohol production, producing desirable volatile compounds, and reducing acetic acid, offering a promising strategy for optimizing regional wine fermentation. Peiris et al. round out this theme with an overview of the *Macrocybe* genus, highlighting its agricultural and economic potential. These giant mushrooms can be cultivated on lignocellulosic agricultural waste, transforming low-value byproducts into nutrient-rich food sources with demonstrated medicinal properties, thus contributing to a circular bioeconomy.

Finally, the Research Topic acknowledges the critical importance of biodiversity as the bedrock of all applied mycology. Two contributions from Karunarathna et al. focus on the taxonomic and biotechnological exploration of fungal diversity in specific, understudied regions. The first reviews the current status, research, and development of the Ganodermataceae family in the Lower Mekong Basin, a biodiversity hotspot. It emphasizes the region’s rich but poorly characterized diversity of these medicinally and economically valuable fungi, underscoring the need for robust taxonomic frameworks to underpin conservation and sustainable utilization. The second (Karunarathna et al.) provides a comprehensive overview of the *Fomitopsis* genus, integrating traditional medicinal knowledge with modern scientific advances to map its biomedical and industrial relevance. These studies serve as a powerful reminder that the discovery and preservation of novel fungal species, through the maintenance of living culture collections and the exploration of unique ecological niches, is not a mere academic exercise but a direct investment in the future pipeline of biotechnological innovation.

In conclusion, this Research Topic presents a compelling cross-section of contemporary mycological research, demonstrating a field in dynamic transition. The contributions collectively illustrate a trajectory from elucidating fundamental regulatory mechanisms, such as those governed by AcuK/AcuM, to the sophisticated engineering of fungi for cannabinoid production and oral drug delivery. They showcase the power of environmental modulation to enhance therapeutic compound yields and highlight the indispensable role of fungal biodiversity as a source of novel solutions. Whether through the immunomodulatory exopolysaccharides of forest basidiomycetes, the dye-decolorizing enzymes of freshwater ascomycetes, or the bioinsecticidal spores targeting disease vectors, fungi are unequivocally proving to be treasured giants. The findings assembled here not only advance our scientific understanding but also lay a robust foundation for translating fungal potential into tangible applications that can address critical global needs in healthcare, environmental remediation, and sustainable industry. The continued exploration and harnessing of this remarkable kingdom remain not just an opportunity but an imperative for a sustainable and healthy future.

